# Hepatocytes express the antimicrobial peptide HBD-2 after multiple trauma: an experimental study in human and mice

**DOI:** 10.1186/s12891-017-1458-8

**Published:** 2017-03-07

**Authors:** Stefanie Fitschen-Oestern, Matthias Weuster, Sebastian Lippross, Peter Behrendt, Sabine Fuchs, Thomas Pufe, Mersedeh Tohidnezhad, Andreas Bayer, Andreas Seekamp, Deike Varoga, Tim Klüter

**Affiliations:** 1 Department of Trauma Surgery, University Medical Center of Schleswig-Holstein, Campus Kiel, Arnold-Heller-Strasse 3, 24105 Kiel, Germany; 2 Department of Trauma Surgery, University of Aachen, Pauwelsstrasse 30, 52074 Aachen, Germany; 3 Department of Cardiovascular Surgery, University Medical Center of Schleswig-Holstein, Campus Kiel, Arnold-Heller-Strasse 3, 24105 Kiel, Germany

**Keywords:** Antimicrobial peptides, Multiple trauma, Human beta-defensin, Liver, Hepatocytes, MBD

## Abstract

**Background:**

Human-beta defensins (HBD) belong to the family of acute phase peptides and hold a broad antimicrobial spectrum that includes gram-positive and gram-negative bacteria. HBD are up-regulated after severe injuries but the source of posttraumatic HBD expression has not been focused on before.

In the current study we analysed the role of liver tissue in expression of HBD after multiple trauma in human and mice.

**Methods:**

HBD-2 expression has been detected in plasma samples of 32 multiple trauma patients (ISS > 16) over 14 days after trauma by ELISA. To investigate major sources of HBD-2, its expression and regulation in plasma samples, polymorphonuclear neutrophils (PMN) and human tissue samples of liver and skin were analysed by ELISA. As liver samples of trauma patients are hard to obtain we tried to review findings in an established trauma model. Plasma samples and liver samples of 56 male C57BL/6 N-mice with a thorax trauma and a femur fracture were analysed by ELISA, real-time PCR and immunohistochemistry for murine beta defensin 4 (MBD-4) and compared with the expression of control group without trauma.

The induction of HBD-2 expression in cultured hepatocytes (Hep G2) was analysed after incubation with IL-6, supernatant of Staphylococcus aureus (SA) and Lipopolysaccharides (LPS). One possible signalling pathway was tested by blocking toll-like receptor 2 (TLR2) in hepatocytes.

**Results:**

Compared to healthy control group, plasma of multiple traumatized patients and mice showed significantly higher defensin levels after trauma. Compared to skin cells, which are known for high beta defensin expression, liver tissue showed less HBD-2 expression, but higher HBD-2 expression compared to PMN. Immunhistochemical staining demonstrated upregulated MBD-4 in hepatocytes of traumatised mice. In HepG2 cells HBD-2 expression could be increased by stimulation with IL-6 and SA. Neutralization of HepG2 cells with αTLR2 showed reduced HBD-2 expression after stimulation with SA.

**Conclusion:**

Plasma samples of multiple traumatized patients showed high expression of HBD-2, which may protect the severely injured patient from overwhelming bacterial infection. Our data support the hypothesis that liver is one possible source for HBD-2 in plasma while posttraumatic inflammatory response.

## Background

Multiple trauma is still the most frequent cause of death in people under 40 years. Due to direct exposure of soft tissue and bone to bacteria in case of open fracture and soft tissue damage multiple traumatized patients are highly susceptible to infectious complications [1, 2]. Furthermore severe injuries lead to a depression of the immune system [3] which favours infectious complications [4].

In previous studies we could demonstrate, that host-defence peptides (HDP) are induced in plasma of multiple injured patients and provide extended antimicrobial capacity [5].

Such peptides constitute an innate defence mechanism that has been shown to be present in various kinds of tissue like skin, lung, brain, stomach and bladder [6–8]. HBDs generally display potent activity against gram-positive as well as gram-negative bacteria, virus and fungi [9]. Additionally defensins and cathelicidins activate T- and B-cells, support wound healing and angiogenesis and induce the attraction of neutrophils [10–13]. So far the sources of HDP in plasma of trauma patients have not been identified yet. In contrast to that many sources of inflammatory mediators and cytokines that contribute to the posttraumatic inflammatory reaction have been described several times [14]. Liver tissue has been attributed with a special role in the release of such mediators [15, 16]. Large amounts of IL-6 are locally produced in the liver by Kupffer cells. Following major trauma the IL-6 release correlates with injury severity and mortality [17]. IL-6 is also known as a main inducer of HBD-2 in vitro and can potentiate the microbial killing activity of HBD-2 [18].

With regard to the potential of HDP in treatment of multiple injured patients it is indispensable to discover HDP producing cells and determine possible pathways for its regulation.

The aim of this study was to detect possible sources for HDP in plasma of multiple injured patients and to find inducers and mechanisms of induction. As liver constitutes an organ, which is vulnerable to trauma damage and additionally plays a major role in the posttraumatic inflammatory response, we focus on liver tissue and compared this to skin as a well know producer of HBD-2. Due to the fact that human liver tissue of multiple injured patients is hardly available for research, we investigated the regulation of murine beta defensin 4 (MBD-4) [19], which is comparable to the human HBD-2, in liver of an established multiple trauma mouse model.

## Methods

### Patient population

Thirty two adult patients with multiple injuries (aged 18–65 years, male and female) were studied at the Department of Trauma Surgery of the University Medical Center of Schleswig-Holstein, Campus Kiel and the Hannover Medical School, Germany. Data were analysed retrospectively. Inclusion criteria were an injury severity score (ISS) of greater than 16 points without the presence of severe traumatic brain injury (though abbreviated injury scales for head injury were included in the ISS) and primary admission to the institutions. Exclusion criteria were penetrating trauma and no direct admission to either institution following the traumatic event. All procedures were in concordance with the Revised Version of the Declaration of Helsinki. The Ethics committee approval was obtained prior to the study for trauma patient as well as for volunteer (control group) sample analysis (AZ: D 415/08).

### Control group

A group of 6 volunteers (3 women and 3 men), age 20 to 40 years, was randomly selected for determination of the normal baseline values. Inclusion criteria were no previous diseases, no medication or trauma and no previous operations.

### Collection of blood

Venous EDTA-blood (9 ml Monovette, Sarstedt, Germany) was collected daily at 7 a.m. from traumatized patients over a 14-day period. The first sample was drawn directly after admission to the hospital. Within 30 min the blood was centrifuged for 10 min at 2000 g at room temperature. The supernatant plasma was stored at −80 °C.

### Recruitment of tissues

Epidermis was collected from 5 patients undergoing osteosynthesis after fracture. After skin incision a 50 × 2 mm piece of epidermis was cut for further examinations. Liver tissue was harvested from 5 patients undergoing liver resection. 10 mg of macroscopic healthy tissue was cut for further examination. All operations were performed at the Departments for Trauma Surgery and Visceral Surgery at the University Medical Centre of Schleswig-Holstein, Kiel Campus. The local ethics committee approval was obtained for the study (AZ: D412/08).

### Isolation of PMN with polymorph-prep©

Venous blood samples of 9 mL were taken from 5 healthy volunteers by the use of EDTA Monovettes. PMN were isolated from whole blood using Polymorph-Prep© (Axis-Shield, Norway) according to the manufacturer’s instructions. 5 mL of blood was carefully layered on 5 mL of Polymorph-Prep©. After centrifuging at 500 × g for 30 min at room temperature mononuclear cells and polymorphonuclear cells were separated and resuspended in 0.45% NaCl.

### Cultivation and stimulation of immortalized liver cells

The experiments were performed with immortalized liver cells, HepG2 (Cell Lines Service, Germany). These cells represent histological and biological characteristics of differentiated parenchymal liver cells [20].

Cells were cultivated as described in distributer’s instructions. For cultivation 800,000 cells were filled into a 25 cm^2^ cell culture bottle with 2 mL of cell medium. Cells were stimulated with 50 ng/mL IL-6 (Tebu, Offenbach, Germany) and supernatant of SA (1:100, assembled as described by Gläser et al. [21]).

For neutralisation experiments, 400,000 cells were cultivated, incubated with 10 $\mu$g/mL TLR2 antibody (eBioscience, San Diego, USA) for 1 h and stimulated afterwards with SA (1:100). Incubation was performed at 37 °C in ambient humidity with 5% CO_2_ in an incubator. All experiments were accomplished three times.

### HBD-2 ELISA

Ninety six-well immunoplates (MaxiSorp, Nunc, Roskilde, Denmark) were coated at 4 °C for 24 h with 100 μl of capture antibody. The antibodies were diluted in 0.05 M carbonate buffer, pH 9.6. Afterwards the wells were blocked with 200 μl of 1% bovine serum albumin in PBS for 60 min at room temperature. After three times washing with 250 μl of PBS + 0.05% Tween 20, 100 μl per well of sample was incubated for 60 min at room temperature. Plates were washed three times with PBS + 0.05% Tween 20 and wells were incubated for 30 min at room temperature with 100 μl of biotinylated antibody diluted in 10 ml 0.1% BSA + PBS. Plates were again washed three times with PBS + 0.1% Tween 20 and filled with 100 μl/well of Streptavidin-HRP (R&D Systems, Minneapolis, USA) diluted 1:200 in PBS + 0.05% Tween 20. The plates were incubated for 30 min at room temperature, washed three times as described above and incubated with 100 μl 3.3′,5.5′-Tetramethylbenzidine (Sigma-Aldrich, St. Louis, USA) as the development agent for 5–10 min at room temperature in the dark. Absorbance was measured at 450 nm with a multichannel photometer (Sunrise; Tecan, Crailsheim, Germany). For the ELISA experiments three wells of each sample were analysed.

For HDB-2 ELISA goat anti-HBD-2 antibody (Acris, Hiddenhausen, Germany; PP1125P2) and biotinylated goat anti-HBD-2 antibody (Acris, Hiddenhausen, Germany; PP1125B2) were used in the concentration 0.5 and 0.25 μg/ml. Human recombinant HBD-2 was (Acris, Hiddenhausen, Germany) served as the standard with the following concentrations: 0, 0.05, 0.1, 0.2, 0.39, 0.78, 1.56, 3.13, 6.25 ng/ml. Experiment were accomplished three times.

### Immunohistochemistry

After fixation of human liver samples in 4% paraformaldehyde, the tissue was embedded in paraffin, sectioned and dewaxed. Endogenous peroxidases in tissue sections were blocked with 3% H_2_O_2_, and tissue sections were subsequently incubated with normal serum (1:5 in Tris buffered saline (TBS)) from the species in which the primary antibody was raised. Immunohistochemical staining was performed on 6 μm paraffin sections, using polyclonal primary antibody against HBD-2 (Goat anti Human Defensin beta 2, diluted 1:200, Acris). Incubation with primary antibody was performed at 4 °C for 12 h. Incubation with second antibody against HBD-2 (Polyclonal Antibody to Goat IgG, diluted 1:300, Acris) was performed for 45 min and incubation with streptavidin-peroxidase for 30 min. Afterwards washing for 5 min was conducted three times. Samples were incubated with DAB (6 mg of 3,3′-Diaminobenzidine tetrahydrochloride in 10 ml distilled water with 2 drops of 3% H_2_O_2_) as chromogen. After washing three times with TBS for 5 min cell nuclei were counterstained with Mayers-Haemalaun for 15 s followed by rinsing of samples for 5 min in distilled water. Samples were dehydrated with different concentrations of alcohol (50, 75, 90 and 95%) and after 5 min covered in Xylol with DPX. Negative control was carried out while dispensing with primary antibody.

### Animal care

Ethical approval has obtained from Ethics Committee of the MHH (Medizinische Hochschule Hannover) (AZ: V 312–72241.121-9 (25-2/11)). The study was performed at the experimental trauma surgery laboratory of the MHH. Experiments were conducted in an operating room at the animal research facility. 56 male C57BL/6 N-mice (Charles River, Germany) weighing 22 ± 3 g were used for the study. 20 mice were used in preliminary experiments to determine the weight needed for induction of chest trauma.

All mice were handled at room temperature for 14 days before treatment and all mice were at the same age of 12 weeks. Throughout the study period, pelleted mouse chow and water were available *ad libitum*. The lighting was maintained on a 12-h light-dark cycle. Analgesic treatment was administered to all animals (200 mg/kg metamizole sodium (Novalgin®, Hoechst, Unterschleißheim, Germany)) throughout the study. All surgical procedures were performed under deep anesthesia with isoflurane (Baxter AG, Volketswil, Germany) and local application of xylazine (16 mg/kg) (Rompun®, Bayer, Leverkusen, Germany). The mice were warmed to 36 °C using infrared heat lamps after the surgical procedures were complete. Wound closure was performed before recovery from the anaesthesia.

### Group distribution and experimental procedures

Two different groups (*n* = 8) were included in the experimental design: a) mice with a femur fracture and chest trauma and b) mice with an intramedullary pin but without chest trauma or femur fracture (control group). The experimental design of the multiple trauma model is based on a two-hit model. The first hit consisted of a closed femur fracture on the right side with the femur stabilized with a cannula before the fracture was performed. The method used for the femur fracture model was first described by Bonnarens (35). After primary wound closure, standardized femur fracture was induced using a blunt guillotine device weighing 500 g (0.784 J). This resulted in a femoral fracture combined with soft tissue injury. Chest trauma immediately followed femur fracture as the second hit. The chest trauma model was previously described for rats (36; 37). Trauma was induced in anesthetized mice by dropping a hollow aluminum cylindrical weight (300 g) through a vertical stainless steel tube onto a Lexon platform resting on the chest. The impact energy E (1.617 J) of the falling weight was calculated using the equation E = m x g x h where m = mass of aluminium weight (in kilograms), g = gravitational acceleration (9.8 ms^−2^) and h = height of weight above the Lexon platform (in meters). Calculations assumed that all the potential energy of the weight was transferred to the animal, neglecting frictional dissipation. The platform was suspended on Teflon guides to minimize friction and facilitate energy transfer to the anesthetized animal. The shield was reproducibly placed entirely over the chest without intrusion onto the abdomen. Animals were sacrificed immediately after trauma, after 6, 12, 24 h, 3 and 7 days and samples for histologic examination were obtain afterwards.

### Protein analysis of MBD-4

Blood samples obtained by heart puncture of mice were centrifuged for five minutes. The supernatant was removed and stored at −20 °C until processed. Concentrations of MBD-4 in plasma samples were analysed by Luminex assay according to standard protocols using LiquiChip200 (Qiagen). A MBD-4 Elisa kit (ABIN415726, antibodies online GmbH) was used for protein detection. Every experiment was accomplished three times.

### Immunhistology/Liver tissue of mice

Six Liver tissue samples of mice were embedded in paraffin. Sections (5 μm) were cut out of the central portion of the liver with a sliding microtome (HM 430; Microm International). They were placed onto Superfrost Plus microscope slides (Thermo Scientific) and left overnight at 60 °C. Sections were routinely stained with hematoxylin and eosin (H&E). Safranin O staining was carried out for 6 min, using a 0.1% aqueous solution at pH 3.0.

### Real-time PCR

For real-time polymerase chain reaction (PCR), RNA was isolated from mice liver tissue with the RNeasy-Total RNA Kit (Qiagen, Hilden, Germany) according to the manufacturer’s instructions. Total RNA (1,000 ng) was used for reverse transcription and subsequent Real-time PCR with gene-specific primers. Real-time PCR was performed as described by Varoga et al. [22]. The gene of interest was detected using TaqMan probes: Mm00731768_m1 (Applied Biosystems, Foster City, CA, USA).

### Statistical analysis

Data is expressed as the mean ± SD of tested samples. Statistical significance was evaluated using one-way ANOVA with Bonferroni correction. Statistical significance was assumed where probability values of less than 0.05 were obtained. Spearman’s linear regression analysis was performed to evaluate correlation of protein plasma concentrations.

## Results

### HBD-2 concentration in plasma samples of multiple trauma patients

**Fig. 1:**
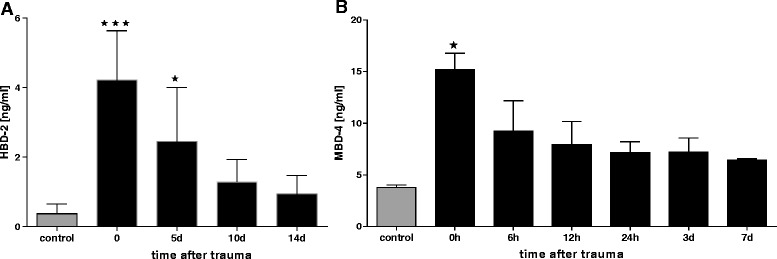
Concentration of host defence peptides in plasma of humans and mice after multiple trauma. **a** ELISA analysis shows HBD-2 in plasma samples of multiple traumatized patients from day 0, day 3, day 7 and day 14 after multiple trauma. Expression was compared to the expression of a healthy control group. **b** In trauma mouse model significant induction of MBD-4 was detected directly after hit. Analogue to human the highest levels were measured on day of trauma. *** = *p* < 0.001; * = *p* < 0.05 versus controls; d = days; h = hours

First we analysed plasma of multiple injured patients and as a control plasma of healthy donors for HBD-2 retrospectively. On the day of trauma protein levels increased 6 times compared to the healthy control group (0.65 ng/ml ± 0.31) to a maximum concentration of 4.2 ng/ml ± 1.7 (Fig. 1a). On day five HBD-2 expression decreased compared to day of trauma but was still significantly higher than the control group. Subsequent levels decreased, but did not reached levels of healthy donors on day 14.

### MBD-4 concentration in plasma samples of mice after multiple trauma

Host defence peptides in plasma of multiple traumatised mice were evaluated directly after trauma until day 7. MBD-4 levels increased significantly to 15.18 ± 2.2 ng/ml on day of trauma compared to control group (3.79 ± 0.5 ng/ml; Fig. 1b). MBD-4 levels decreased afterwards, but did not reach the concentrations of control until day 7.

### HBD-2 concentration in samples of lysed liver tissue, skin and PMN

**Fig. 2:**
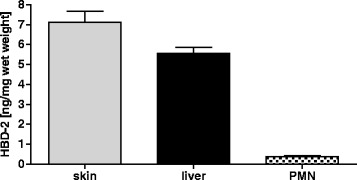
Analysis of different sources for HBD-2 in human plasma. Samples of skin, liver and PMN of healthy donors were tested by Elisa analysis. Human liver samples contain similar concentrations of HBD- 2 compared to skin. Neutrophil granulocytes hold lowest levels

To evaluate possible sources of HBD-2 in human plasma we analysed liver and PMN, whether they contained comparable protein concentrations as skin, a well-known producer of HBD-2 (Fig. 2). As expected skin samples contained the highest concentration of HBD-2 (7.12 ng/mg wet weight ± 0.5). Liver tissue lysate showed less concentration of HBD-2 compared to skin (5.55 ng/mg wet weight ± 0.5), but higher concentration compared to PMN (0.3 ng/mg wet weight ± 0.5).

### Immunohistochemestry of host defence peptides in liver tissue of humans and multiple trauma mice

**Fig. 3:**
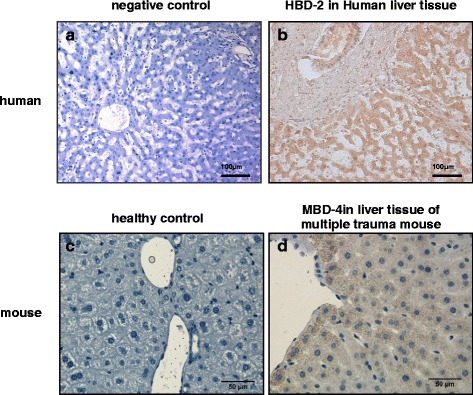
Host defence peptides in liver tissue of human donor and multiple traumatised mice. **a** and **b** Immunohistochemistry reveals expression of HBD-2 in healthy human liver tissue. Negative control studies were carried out by absorption of the primary antibody by recombinant protein (1:500 dilution). **c** and **d** MBD-4 was stained in liver tissue samples of day 1 after multiple trauma. Compared to control, posttraumatic liver samples of mice show high immunoreactivity for MBD-4

Immunohistochemistry was performed in liver specimens of non-trauma patients to confirm the detection of HBD-2 (Fig. 3a + b). Human liver samples showed strong positive staining for HBD-2. Mice liver of animals with multiple trauma revealed positive MBD-4 staining, whereas control liver showed no reactivity (Fig. 3c + d).

### Gene expression of MBD-4 in liver samples of multiple trauma mice

**Fig. 4:**
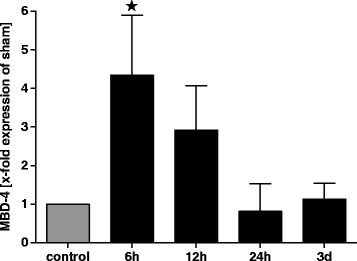
Elevation of MBD-4 in liver cells of mice after multiple trauma. Induction of MBD-4 in liver of traumatised mice over a period of 3 days detected by real-time PCR. Significant elevation of MBD-4 can be detected after 6 h after trauma compared to control group. * = *p* <0.05

Increase of MBD-4 in mice liver after multiple trauma was verified by real-time PCR. Analogue to plasma levels expressions raised after 6 h (4.3-fold of sham ± 3.4) and 12 h (2.9-fold of sham ± 2.3) up to 5.0-fold of sham expression. The MDB-4 expression reached sham levels after 24 h (1.0-fold of sham ± 0.5) (Fig. 4).

### Induction of HBD-2 in immortalized hepatocytes

**Fig. 5:**
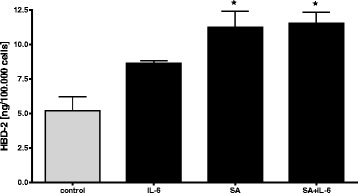
In vitro stimulations of HBD-2 in Hep G2 cells. Human immortalized liver cell (Hep G2) were stimulated with bacteria and proinflammatory cytokines and concentrations of HBD-2 in lysates were analysed by ELISA. Significant up-regulation of HBD-2 after stimulation with SA and SA + IL-6 were detected in HepG2 cells. * = *p* < 0.05 versus control

To evaluate induction of HBD-2 in immortalized hepatocytes (Hep G2), cells were stimulated with IL-6 (8.6 ng/ml ± 0.1), Staphylococcus aureus (11.2 ng/ml ± 1.1) and Staphylococcus aureus in combination with IL-6 (11.5 ng/ml ± 0.8). Lysates were tested by Elisa (Fig. 5). HBD-2 induction increased compared to control (5.2 ng/ml ± 1) but interestingly stimulation with SA and IL-6 did not cause a higher HBD-2 expression compared to stimulation with SA.

### Role of TLR2 in HBD2 induction in Hep G2 cells

**Fig. 6:**
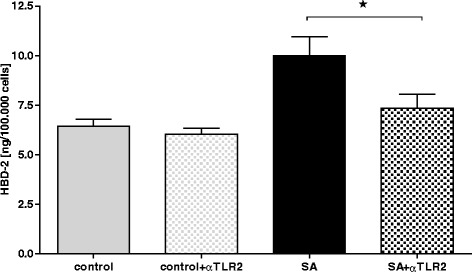
Role of TLR2 in HBD-2 induction in Hep G2 cells. TLR2 was blocked with Antibodies (αTLR2) and stimulated with SA afterwards. The induction of HBD-2 in immortalized liver cell culture was measured by Elisa after stimulation. Compared to sample with blocked TLR2 a significant increase can be seen for HBD-2 after stimulation with SA. * = *p* < 0.05 SA versus SA + αTLR2

HBD-2 induction is regulated by toll-like receptors, especially TLR2. Hep G2 were incubated with TLR2 blocking antibody (αTLR2), SA and SA together with αTLR2 (Fig. 6). After neutralisation with αTLR2 HBD-2 concentration was slightly decreased compared to control levels (7.3 ng/ml ± 0.9). The increase of HDP after treatment with SA + αTLR2 was significantly lower compared to stimulation with SA alone. Significant HBD-2 up-regulation could be shown after stimulation with SA (10.0 ng/ml ± 1.3).

## Discussion

Due to extended wounds and open fractures multiple injured patients are exposed to a multiplicity of invading gram-positive and -negative bacteria. Regarding this precarious premises one might believe that the overall sepsis rate is relatively low [2, 23]. In a previous study we were able to demonstrate that plasma of multiple injured patients provides an increased antimicrobial activity compared to healthy individuals [5]. In addition we detected HDP with its direct ability to invade microbes as possible effectors for the antimicrobial capacity. Nevertheless a possible source for the HDP and possible regulating pathways have not been investigated yet.

The liver is known as a major source of acute phase proteins, which are recognized as important components of the innate immune system [16, 24]. In the current study we could show that liver tissue contains similar amounts of HBD-2 as skin tissue, which is recognized as a main producer for HBD [25]. Therefore we analysed the HBD-2 expression in liver tissue immunohistochemically and could detect hepatocytes as the main producer. Due to the fact that human hepatocytes of multiple injured patients is hardly available for research, we had to apply an established multiple trauma mouse model [26]. MBD-4 constitutes an inducible orthologues of HBD-2 and holds strong antimicrobial activity as well [27].

In the present study we were able to show, that the time course of MBD-4 levels in plasma of trauma mice has a similar trend to the HBD-2 levels in plasma of multiple injured patients. Highest levels of MBD-4 were detected immediately after trauma, decreased afterwards and even after 7 days plasma concentrations did not reach the concentration of the heathy mice.

In further investigations we demonstrated that MBD-4 expression is induced in liver tissue after multiple trauma. The plasma concentrations of MBD-4 correlated with their expression in liver. These findings were supported by our immunohistochemical staining of liver tissue from traumatised mice, in which we determined higher MBD-4 concentrations compared to liver of a healthy mice. It can be assumed that liver is one source for HDP in plasma after multiple injury.

Hypovolemic shock, hepatic ischemia and reperfusion injury as it occurs in multiple trauma patients stimulate the production of reactive oxygen species and the release of cytokines [28]. To get one step forward to comprehend the regulation of HBD-2 after multiple trauma we stimulated liver tissue with cytokines and gram-positive bacteria. As shown before bacteria seem to be a strong inducer for lysozyme in different tissues [29]. With regards to open fracture, wounds and deep tissue contusion, bacteria are a major cause for systemic infection after trauma. Our in vitro data revealed that SA induce HBD-2 in liver. Tissue damage seems to be another initiator of posttraumatic inflammatory reaction and besides the degree of tissue damage correlates with release of pro-inflammatory cytokines like IL-6 [30–32]. Due to the fact that IL- 6 plays a major role in posttraumatic inflammatory reaction [33], we tested the key player for its effect on HBD-2 expression in liver tissue. IL-6 shows non-significant induction of HBD-2 in liver tissue and hepatocytes, whereas SA seems to be the strongest inductor. HBD-2 induction by gram-positive bacteria has been reported in epithelial cells but not in hepatocytes [34, 35].

Relating to systemic inflammation and shock syndrome, liver constitutes a key figure in terms of the regulation of mediators (28) and regulate innate defence after severe injury (29).

To extend the comprehension of HBD-2 regulation in hepatocytes we identified one signalling pathway in hepatocytes. As shown before in epithelial cells TLR2 is involved in the regulation of HBD-2 [36, 37]. Furthermore TLR constitute the major mediators of inflammatory responses in liver and recognize microbial components as well as endogenous ligands from damaged or stressed cells [38, 39].

Toll-like receptors promote proinflammatory signalling such as the nuclear factor-κB, c-Jun-N-terminal kinase (JNK), p38, and interferon pathways in the liver and regulate antiviral and antibacterial responses, hepatic injury, and wound healing [39]. In the present study we could demonstrate, that after blocking TLR2 with neutralizing antibodies, stimulation of hepatocytes with SA showed no effect. We assume that TLR 2 is not only involved in HBD-2 regulation in epithelial cells, but also in hepatocytes.

## Conclusion

For the first time we provide evidence, that the liver is involved in the regulation of HBD-2 after multiple trauma. With the current study we increase the understanding of the complex immune reaction after severe injury. We are well aware of the fact that there are other sources for HBD-2 which can be recruited in case of trauma. Furthermore it can be assumed that there exists a multiplicity of other HDP and other features of this heterogenic group with respect to multiple trauma and critical care. Considering the complexity of this issue, the role of HDP in trauma will require further attention.
